# Initial Clinical Practicum Stress among Nursing Students: A Cross-Sectional Study on Coping Styles

**DOI:** 10.3390/ijerph18094932

**Published:** 2021-05-06

**Authors:** Eunhee Hwang, Mijung Kim, Sujin Shin

**Affiliations:** 1 Department of Nursing, Wonkwang University, Iksan 54538, Korea; ehh@wku.ac.kr; 2 Department of Nursing, Kyungil University, Gyeongsan 38428, Korea; mjkim@kiu.kr; 3 College of Nursing, Ewha Womans University, Seoul 03760, Korea

**Keywords:** nursing students, clinical practicum, psychological stress, coping

## Abstract

Nursing students experience various stressors during their initial clinical practicum. As these stressors negatively affect learning and performance, coping strategies are essential. Therefore, this research study explored the relationship between coping styles and stress levels using a cross-sectional study with a convenience sample of 184 nursing students. Clinical practicum stress and coping styles were assessed via electronic questionnaires, and the data were analyzed using descriptive statistics, t-tests, and variance analyses. The highest score for clinical practice stress was for the practical education environment and practical work burden. The total stress score differed significantly according to coping style (t = −2.36, *p* = 0.020), and the total stress score of the passive coping group was higher. Among the sub-categories of stress, the scores of the education environment (t = −2.68, *p* = 0.008) and having undesirable role models (t = −2.14, *p* = 0.034) were significantly higher in the passive coping group. Although practical work burden was the highest stress factor in the active coping style group, the stress on the environment was highest in the passive coping group. The findings show that professors and clinical educators should recognize the various coping styles and incorporate different teaching methods in the clinical setting.

## 1. Introduction

Nursing education is structured in such a way that learning through experience is achieved by applying theoretical knowledge and skills to clinical practice with patients [1]. Nursing students can develop their professional characteristics and clinical judgment through clinical experiences and also have the opportunity to develop critical thinking, confidence and relationship-building skills [2]. It is important to close the gap between theory and the field through clinical practicum experience. In addition, nursing students integrate nursing knowledge and practice through clinical practicum education [3]. To become a successful professional nurse after graduation, effective clinical practice is fundamental.

Previously identified clinical sources of stress include lack of knowledge and professional ability, lack of familiarity with history and medical terms, a heavy workload, being in an unfamiliar situation, making mistakes with patients and handling technical instruments [4,5,6]. Stress in education is an important issue because it interferes with learning and performance [6]. A systematic review of the sources of nursing students’ stress [7] emphasized the need for effective coping strategies to mediate stress in nursing education and clinical practicum.

In particular, nursing students entering clinical practicum training for the first time have more difficulty during the initial clinical practicum because of a lack of experience. For example, a previous qualitative study revealed that nursing students were not satisfied with their clinical experiences and found their initial clinical experiences to be very stressful [8].

Folkman and Lazarus categorized coping methods into two distinct categories: emotion-focused and problem-focused [9]. Emotion-focused coping aims to reduce, control and manage negative emotional reactions that arise as a result of a stressful experience (i.e., that are not a cause of stress). However, problem-focused coping is directed toward reducing stress by targeting the root causes of stress. In other words, problem-focused coping can be seen as active coping, whereas emotion-focused coping is seen as passive coping. Students’ stress in their clinical practice can be altered and influenced by the coping strategies they choose to employ. As well as helping students to perform better in their studies, coping strategies can also help to relieve students’ stress [10].

Numerous studies have identified the levels of stress and coping strategies used by nursing students. Studies have shown that nursing students experience moderate levels of stress and use more positive coping strategies than negative strategies [11,12]. However, one study showed that the passive coping strategy was used more frequently by nursing students as their perceived stress levels increased [13]. Evidence related to the stress of nursing students during their initial clinical practicum and their stress-coping strategies remains limited. Understanding the impact of stress related to clinical practicum and having proper coping mechanisms is invaluable for both nursing students and educators as it could promote the development of an effective clinical teaching strategy for nursing education.

This study was designed to examine the stress and coping styles of nursing students who completed the initial clinical practicum. The purposes of this study are (1) to describe stress related to clinical practicum and stress-coping styles during the first clinical practicum and (2) to identify the differences in stress related to clinical practicum according to different coping styles.

## 2. Materials and Methods

### 2.1. Design

This study adopted a descriptive design to investigate nursing students’ stress according to coping styles during their first clinical practicum.

### 2.2. Participants

The subjects of this study were college nursing students who had completed their initial clinical practicum in university nursing programs. The inclusion criteria were nursing students who had participated in the first clinical practicum for at least 6 weeks within the last 6 months. A G * Power 3.1.9.2 (Heinrich-Heine-Universität, Düsseldorf, Germany) analysis was conducted to predict the sample size required for a regression analysis with an effect size of 0.15, significance level of 0.05 and power of 0.95, which was 172. Data from 191 subjects were collected to account for an expected dropout rate of 10%. After recruiting 191 subjects, data from 184 were included in the analysis (7 were excluded as their types of stress coping were not clearly identified).

### 2.3. Data Collection and Ethical Considerations

This study was approved by the Institutional Review Board (IRB) (Ewha-201906-0010-01). Data were collected using an Internet-based electronic survey from the end of June 2019 to the beginning of July 2019, which was when the initial clinical practicum was completed. To investigate the overall stress of clinical practice in the first semester rather than the stress at a specific practicum point in time, a survey was conducted at the time when the first semester’s clinical practice was completed. The advantages of using Internet-based online surveys include access to individuals in distant locations, overcoming the difficulty of contacting participants, and the convenience of having automated data collection, which reduces the time and effort required of researchers [14]. For this study, nursing students received an invitation e-mail containing the URL of the web survey, and participation was voluntary. The subjects who participated in this study were considered vulnerable as they were students of the institution to which the researcher belonged. When collecting data from these specific students, a researcher that was not affiliated with the same institution contacted a student representative and explained the purpose of the research to ensure voluntary participation and anonymity of responses. Students were informed that their participation would not be graded and there would be no penalties for choosing not to participate. Following this, informed consent was obtained from all voluntary participants.

### 2.4. Measures

The questionnaire consisted of 94 items, including 8 general characteristics such as sex, age, perceived interpersonal relationship, grade point average, mentor presence, major satisfaction, clinical practice satisfaction and intention to change one’s major. It also included 24 questions about clinical practice stress and 62 questions about stress-coping styles.

#### 2.4.1. Stress Related to Clinical Practicum

Stress related to clinical practicum was measured using a tool comprising 24 items, which was originally developed by Beck and Srivastava [15] and later modified by Kim and Lee [16]. The tool consisted of the following questions: five practical education environments (e.g., “There is not enough space and facilities for clinical practice”), six undesirable role models (e.g., “There are times when nurses show off-principle behavior in nursing practice”), four practical work burdens (e.g., “There are situations in which too many tasks are charged during practice”), four interpersonal conflict issues (e.g., “I have often seen poor relationships with nurses and other medical personnel”; “There are many conflicts and disagreements with friends of the same training group”), and five conflicts with patients (e.g., “It is often difficult to establish a relationship with the patient”; “I have experience practicing in the presence of a hostile patient”). Each question was answered on a 5-point Likert scale ranging from 1 (strongly disagree) to 5 points (strongly agree), whereby a higher score indicated higher clinical practicum stress. The internal reliability was 0.92.

#### 2.4.2. Stress-Coping Style

For stress-coping styles, a tool that was developed by Folkman and Lazarus [17] and modified by Kim [18] was used. The tool consisted of 62 questions that comprised 2 subscales: active coping style (e.g., “I analyze it in detail to better understand the problem”; “I tell a person what I am feeling”) and passive coping style (e.g., “I want to avoid hanging out with people and be alone”; “I hope it disappears or ends”). Each item was scored on a 4-point Likert scale ranging from 1 point (not applicable) to 4 points (very frequent). In this study, each subject was classified into the type with the highest mean score among the two subscales. This means that higher scores indicate greater use of corresponding coping strategies. After obtaining the scores for the active coping strategy and the passive coping strategy, a coping-style group was assigned to each participant. Although the difference in score for using each coping strategy was small, the coping style with the highest score among the two categories was assigned.

The Cronbach’s alpha coefficients were 0.94 and 0.87 for the active coping style and the passive coping style, respectively.

### 2.5. Data Analysis

The collected data were analyzed using IBM SPSS version 25.0 (IBM Corp., Armonk, NY, USA). General characteristics, clinical practicum stress and stress-coping styles were analyzed using descriptive statistics. To analyze the differences in clinical practicum stress according to stress-coping style, coping styles were divided into two groups (active coping style and passive coping style). The differences in clinical practicum stress were analyzed according to stress-coping styles and general characteristics using a variance analysis (ANOVA) with Least Significant Difference (LSD) post hoc comparison.

## 3. Results

### 3.1. Subjects’ General Characteristics

Most of the subjects were women (91.8%), and the mean age was 22.5 years. There were no statistically significant differences between the two groups in terms of gender, age, having a mentor, satisfaction with their major, satisfaction with clinical practicum and intention to change their major. In the active coping group, the percentage of subjects who reported that their interpersonal relationships were good was 81.7%, while 17.3% reported that they were moderate; in the passive coping group, the percentage of subjects who responded that interpersonal relationships were good was 66.2%, and the percentage of subjects who responded that they were moderate was 30.0%. As such, there were statistically significant differences between the two groups (Table 1).

### 3.2. Clinical Practicum Stress and Stress-Coping Styles

The mean score of clinical practice stress was 3.1 (range: 2–5), and the highest score was 3.5 for practicum education environment and the practical work burden, followed by 3.1 for the undesirable role model, 3.0 for conflict with patients and 2.6 for conflict with patients. In terms of the analysis of stress-coping styles, 104 individuals had a dominant active coping style, whereas 80 had dominant passive coping styles (Table 2).

### 3.3. Differences in Stress Related to Clinical Practicum according to General Characteristics

Table 3 shows the differences in stress related to clinical practicum according to the general characteristics. The total stress score differed significantly according to sex (t = −1.99, *p* = 0.048), satisfaction with clinical practice (F = 16.22, *p* < 0.001) and intention to change majors (t = 2.86, *p* = 0.005). In regard to the general characteristics, other differences in total stress that were related to clinical practicum were not statistically significant.

### 3.4. Differences in Stress Related to Clinical Practicum according to Stress-Coping Styles

Table 4 shows the differences in stress related to clinical practicum according to stress-coping styles. The total stress score differed significantly between the groups (t = −2.36, *p* = 0.020) and the group with a passive coping style had higher stress levels than the active coping group. Among the sub-categories of stress, stress scores related to the practicum education environment and having an undesirable role model were significantly higher in the passive coping group. Stress related to the practical work burden, interpersonal conflicts and conflict with patients were not significantly different (Table 4).

## 4. Discussion

This study investigated whether the level of stress experienced by nursing students during their first clinical practicum varied according to the primary coping style used by the students. In addition, the reason for understanding the impact of clinical practicum stress on students and understanding the proper way of coping with future clinical practicum courses was presented.

Overall, the clinical practicum stress for this study’s subjects was moderate, which was slightly higher than the clinical practicum stress seen in students in the last semester before graduation [19]. Similar to our own findings, a systematic review of stress in college nursing students found that most studies reported that nursing students experienced moderate levels of stress [20].

In this study, the nursing students experienced the highest levels of stress related to the practical education environment and practical work burden during the first clinical practicum, and the stress level related to the undesirable role model was also high. The clinical setting is an invaluable learning environment for nursing students. However, the learning process that occurs in this environment may also cause students to experience stress [21]. Clinical practicum stress is one of the three major stressors among nursing students, namely academia, clinical practice and personal or social environment [22]. Among the general characteristics of subjects, the factors related to clinical practice stress in this study were satisfaction with clinical practicum and intention to change their majors. This means that higher clinical practicum stress is related to lower satisfaction with clinical practicum and may be a factor that increases the intention of students to change their major. Therefore, clinical practicum stress and clinical practicum satisfaction are major factors for the adaptation and retention of nursing students in college.

Nursing students who experienced their initial clinical practicum reported the following stressors: lack of experience and competence in patient care, situational judgment, being unfamiliar with medical history and medical terminology, low grades of concern, lack of knowledge of patient care required for specific health problems, differences between theory and practice and early starts in the morning (7 a.m.) [11,22]. Therefore, clinical practicum placement must be understood as an opportunity to learn more rather than as a stage of evaluating or testing theoretical knowledge [7]. Clinical practice in nursing education is essential for the transition from nursing students to registered nurses. The quality of supervision, an adjustment to the role of a professional nurse and the comfort, confidence and competence achieved were associated with facilitated transition [23].

According to the analysis of stress-coping styles, there were 104 active coping dominant individuals and 84 passive coping dominant individuals. Passive coping methods were positively correlated with clinical practicum stress [24], indicating that passive coping styles negatively affect stress perception [25]. Students with an active coping style tend to use problem-solving coping strategies, which are negatively correlated with stress [26] and negatively correlated with stress related to patient care and lack of expertise or skills [11]. That is, the degree of stress perception differs depending on the type of stress coping, so a personalized approach is needed to identify and mediate stress factors based on each student’s coping style.

The group with a passive coping style had higher stress levels than the active coping style group. Stress related to practicum education environment and having undesirable role models had higher mean scores in the passive coping style group. This suggests that students with dominant passive coping styles are particularly vulnerable to clinical practicum stress in the first semester practicum. Therefore, how to assign groups to practice with other types of students that have relatively low levels of stress and can complement each other to ensure effective collaboration in clinical practicum ought to be considered. Additionally, in the case of clinical practice in the first semester, it is necessary to create an educational environment that supports learning by matching with preceptors (especially for students who use passive coping strategies) and to reduce the burden of clinical practice.

Furthermore, in both the active coping and passive coping styles, the mean score of the practical work burden was one of the highest stress factors. Meanwhile, stress in the practice education environment was higher in the active coping group. In addition, across the different coping styles, nursing students had the lowest levels of stress related to interpersonal conflict. The perceived learning environment is an important factor in decreasing learners’ stress [27]. These stresses affect physical and psychological symptoms [28] and are related to academic performance [13]. Based on these results, it is necessary to find ways to increase the effectiveness of clinical nursing education through the relationship with practitioners in the clinical field rather than assigning an excessive burden of practical work in the first clinical practicum. Understanding the impact of initial clinical practicum stress on students and efforts to promote positive welcoming experiences for students will ultimately lead them to grow into professional nurses.

This study has some limitations. First, convenience sampling was used, which could limit the generalizability of our findings. Second, students were classified based on the stress-coping type they scored highest in. As such, the potential of students having complex coping styles with similar scores was not considered. Finally, the findings of this study are limited as they describe only association and cannot explain causal relationship.

The novel insight provided by this study is that students using passive coping strategies such as emotion-focused coping and wishful thinking coping have high stress related to clinical practicum. Problem-solving strategies, whereby students maintain an optimistic attitude and adopt various strategies to solve problems, are more effective in reducing stress than emotion-based strategies. Therefore, it is necessary to improve problem-solving capabilities through clinical field observation, pre-practical simulation practice, peer learning and mentoring programs to reduce stress and to encourage the use of active coping methods rather than passive coping during stressful situations. Therefore, it is essential for professors and clinical educators to recognize that students have different stress-coping styles and to incorporate different teaching methods in clinical settings. As such, future studies that investigate the changes in clinical practicum stress ought to explore the group characteristics of practicum students, the underlying reasons for stress during clinical practicum and the differences in coping strategies according to stress sources.

## 5. Conclusions

The results of this study showed that stress related to the first clinical practicum of nursing students was mainly due to the clinical practice education environment and the practical work burden, and the stress level of the clinical practice education environment was higher in the group that had a dominant passive coping style. It is essential that professors and clinical nurse educators recognize students’ stress and facilitate their learning during clinical practicum. In addition, professors and clinical nurse educators should recognize that students have different stress-coping styles. To tackle this, they ought to incorporate different teaching methods in clinical settings, such as creating groups of students that consist of different stress-coping types. This study is meaningful in that it reports on initial clinical practicum stress depending on stress-coping styles and suggests several potential strategies for better clinical education. Based on our findings, we propose that future studies investigate the changes in clinical practicum stress and the underlying reasons for longitudinal stress during clinical practicum.

## Figures and Tables

**Table 1:** General characteristics of nursing students that had completed their initial practicum (*n* = 184).

Variables	Response Categories	*n* (%) or Mean ± SD		t or χ^2^	*p*
		Active Coping Group (*n* = 104)	Passive Coping Group (*n* = 80)		
Sex	Male Female	10 (9.6) 94 (90.4)	5 (6.3) 75 (93.8)	0.68	0.408
Age (years)		22.5 ± 1.64	22.4 ± 0.79	0.80	0.426
Perceived interpersonal relationships	Not good Moderate Good	1 (1.0) 18 (17.3) 85 (81.7)	3 (3.8) 24 (30.0) 53 (66.2)	6.25	0.044 *
GPA reported by subjects	A (≥4.0) B (3.0–3.99) Less than C (<3.0)	17 (16.3) 73 (70.2) 14 (13.5)	15 (18.8) 46 (57.5) 19 (23.8)	3.95	0.139
Having a mentor	Yes No	32 (30.8) 72 (69.2)	22 (27.5) 58 (72.5)	0.23	0.629
Satisfied with their major	Dissatisfied Moderate Satisfied No response	4 (3.9) 32 (31.4) 66 (64.7) 2	9 (11.3) 21 (26.3) 50 (62.4)	3.81	0.149
Satisfied with clinical practicum	Dissatisfied Moderate Satisfied No response	15 (14.7) 29 (27.4) 58 (56.9) 2	15 (18.8) 30 (37.4) 35 (43.8)	3.09	0.213
Intention to change major	Yes No	5 (4.8) 99 (95.2)	6 (7.5) 74 (92.5)	0.58	0.445

GPA, Grade point average; * *p* < 0.05.

**Table 2:** Descriptive statistics of variables (*n* = 184).

Variables	Sub-Categories	Mean (SD) *n* (%)	Min	Max
Stress related to clinical practicum	Total stress score Practicum education environment Undesirable role model Practical work burden Interpersonal conflicts Conflict with patients	3.1 (0.57) 3.5 (0.80) 3.1 (0.71) 3.5 (0.74) 2.6 (0.64) 3.0 (0.69)	2.00 2.00 2.00 2.00 2.00 2.00	5.00 5.00 5.00 5.00 5.00 5.00
Stress-coping style	Active coping Passive coping	104 (56.5) 80 (43.5)		

**Table 3:** Stress related to clinical practicum according to general characteristics (*n* = 184).

Variables	Response Categories	Mean (SD)	t or F	*p*	LSD
Sex	Male Female	2.8 (0.57) 3.1 (0.57)	−1.99	0.048 *	
Perceived interpersonal relationships	Not good Moderate Good	3.2 (0.63) 3.2 (0.52) 3.1 (0.59)	0.25	0.776	
GPA reported by subjects	A (≥4.0) B (3.0–3.99) Less than C (<3.0)	3.3 (0.51) 3.0 (0.57) 3.2 (0.61)	2.64	0.074	
Having a mentor	Yes No	3.1 (0.64) 3.1 (0.54)	−0.03	0.978	
Satisfied with their major	Dissatisfied Moderate Satisfied	3.4 (0.66) 3.1 (0.53) 3.1 (0.58)	1.39	0.251	
Satisfied with clinical practicum	Dissatisfied ^a^ Moderate ^b^ Satisfied ^c^	3.5 (0.57) 3.2 (0.45) 2.9 (0.56)	16.22	<0.001 *	a > b > c
Intention to change major	Yes No	3.6 (0.65) 3.1 (0.55)	2.86	0.005 *	

* *p* < 0.05.

**Table 4:** Stress related to clinical practicum according to stress-coping styles (*n* = 184).

Clinical Practicum Stress	Stress-Coping Style		t	*p*
	Active Coping Style (*n* = 104)	Passive Coping Style (*n* = 68)		
Total stress related to clinical practicum	3.0 (0.58)	3.2 (0.55)	−2.36	0.020
Practicum education environment	3.3 (0.80)	3.6 (0.77)	−2.68	0.008
Undesirable role model	3.0 (0.69)	3.2 (0.72)	−2.14	0.034
Practical work burden	3.4 (0.74)	3.5 (0.74)	−0.86	0.391
Interpersonal conflicts	2.5 (0.59)	2.7 (0.70)	−1.78	0.077
Conflict with patients	2.9 (0.72)	3.1 (0.65)	−1.81	0.073

## Data Availability

The data presented in this study are available upon request from the corresponding author. The data are not publicly available for legal and privacy issues.
